# Looking forward: *Disasters* at 40

**DOI:** 10.1111/disa.12327

**Published:** 2019-02-12

**Authors:** Elena Fiddian‐Qasmiyeh

**Affiliations:** ^1^ Professor in Migration and Refugee Studies University College London (UCL) United Kingdom

**Keywords:** interdisciplinarity, intersectionality, localisation of aid, long‐term responses to disasters, migration, overlapping displacement, refugee‐led response, South–South cooperation

## Abstract

This paper reflects on contemporary studies of and responses to disasters, highlighting the importance of historical, spatial, and intersectional modes of analysis, and draws on the author's ongoing research on Southern‐led and local community responses to displacement in the Middle East. Acknowledging the plurality of ‘international communities of response’, it begins by critiquing the depiction of selected responses to disasters as ‘positive’ ‘paradigm shifts’, including in reference to the ‘localisation of aid, and the United Nations’ Regional Refugee and Resilience Plan for Syria. Next it turns to three key themes that are central to disasters studies: migration; forced displacement; and Southern‐led responses to disasters. Among other things, the paper argues that exploring the principles and modalities of South–South cooperation, rather than promoting the incorporation of Southern actors into the ‘international humanitarian system’ via the localisation agenda, presents a critical opportunity for studies of and responses to disasters.

## Introduction

This paper, based on a keynote lecture presented at the fortieth anniversary conference of the *Disasters* journal in London, United Kingdom, on 14 September 2017, reflects on the current state of studies of and responses to disasters. Among other things, it argues that research, policy, and practice have demonstrated the necessity of looking back (using historical analyses), looking around (using geographically sensitive lenses attentive to scale and space, and by acknowledging the significance of Southern‐led responses), and looking through different lenses (using intersectionalist and interdisciplinary research, and by questioning the locus of one's gaze). From the premise that historical, spatial, and intersectional modes of analysis are essential, the paper takes as one of its starting points that ‘the’ (normative, Northern‐led) ‘international humanitarian community’ is only one of a plurality of ‘international communities of response’, some of which work with, and others explicitly against, ‘the’ hegemonic Northern‐led humanitarian system. It is in part precisely by acknowledging this plurality of ‘communities of response’ across time and space that one can, and must, engage critically with the increasingly mainstream depiction—in official policy discourses and on both policy and political agendas—of selected contemporary responses to disasters as ‘positive’ ‘paradigm shifts’.

The first part of the paper concentrates on the (recurrent) invocation that the international community must interweave short‐term with long‐term responses to disasters, and support multiscalar and multi‐stakeholder responses. With respect to the latter, it discusses the ‘localisation of aid’ and the Regional Refugee and Resilience Plan (3RP) of the United Nations (UN) to respond to the Syria crisis, the latter of which, in particular, emerges in official policy and discourse as one of the quintessential ‘paradigm shifts’ of the twenty‐first century. Building on the theme of interconnected scales of response and analysis, the paper goes on to suggest the importance of responses to mass disasters that centralise rather than postpone engagement with the implications of intersecting identity markers and structures of inequality. As a further example of the importance of exploring intersections in disaster studies, the first part of the paper draws to a close by reflecting briefly on the relationship between research and policy agendas in relation to the UK government's Global Challenges Research Fund (GCRF).

The second part of the paper turns to three key themes that are, and will continue to be, central to disasters studies in the twenty‐first century: migration (including in the context of climate change); forced displacement; and Southern‐led responses to disasters. By examining these processes in a tempo‐spatially‐sensitive manner, the paper warns against the discursive, policy, and/or political framing of migration ‘as’ a crisis, and instead underlines the importance of further research on three under‐evaluated dynamics: immobility; the overlapping nature of forced displacement; and refugee–refugee relationality.^1^ It concludes by focusing on ‘South–South Cooperation’ (SCC) with respect to (or contra) the localisation of the aid agenda,^2^ contending that exploring the *principles* and *dynamics* of SSC, rather than promoting the incorporation of Southern *actors* into the ‘international humanitarian system’ via the localisation agenda, offers a critical opportunity for studies of and responses to disasters.

## Key approaches in contemporary disasters studies and response

### Paradigm shifts, recycling, and the importance of looking back to move forward

It is widely accepted that disasters are not ‘natural’; rather, vulnerability to environmental hazards is framed by economic, political, and social structural factors and processes (*Disasters*, passim). Equally, it is increasingly ‘mainstream’ to acknowledge that it is both insufficient and incorrect to conceptualise disasters as ‘unpredictable’ emergencies that require ‘immediate’ short‐term responses in a ‘humanitarian mode’ that one cannot plan for in advance. As such, it is almost standard for international agencies, donors, and even research council initiatives such as the GCRF (see below) to acknowledge the necessity of interweaving short‐term response elements with long‐term planning. In essence, this entails starting from the principle that development and humanitarian work must be forward‐looking rather than reactive and responsive, such as by identifying and implementing means to reduce risks, as well as to mitigate and plan for and to prevent disasters (see *Disasters* 25(3)). In practice, this often entails integrating longer‐term elements *into* ‘immediate’ scale responses rather than vice versa, thereby ultimately remaining reactive in nature. Nonetheless, policymakers, practitioners, and scholars from across the wide arena of disaster studies acknowledge the need to start from the perspective of long‐term planning and prevention, into which reactive responses can be inserted as and when necessary as anticipated and unanticipated disasters arise.

This mainstreaming is commonly presented as an ‘advance’ vis‐à‐vis international approaches to disasters, providing an essential move away from reactive, emergency‐mode, care, and maintenance approaches.

Even the briefest historical reflection, though, demonstrates that this ‘forward‐looking’ policy and funding agenda is itself a ‘return’ to, or even a ‘recycling’ of, longstanding debates. In the field of refugee response alone, this dates back to at least the 1960s (Crisp, 2001), including in the form of the United Nations High Commissioner for Refugees (UNHCR)'s ‘integrated zonal development approach’, followed by its ‘refugee aid and development’ strategies of the 1980s, the upsurge of interest in the ‘relief‐to‐development’ continuum in the 1990s (Borton, 1994), and the widespread official institutionalisation of diverse ‘development assistance programmes’ in the 1980s and 1990s (UNHCR, 2005). While this is undoubtedly an essential approach with potentially wide‐reaching implications, it is neither an ‘innovation’ nor a paradigm with its origins in the 1990s, as asserted by Hinds (2015), or in the 2000s, as claimed by UNHCR (2005); such forward‐looking approaches at the UN, international non‐governmental organisation (NGO), and donor level have existed for more than 50 years, even if they have been only partially implemented (if at all) (Crisp, 2001). Indeed, over the past few decades, members of the ‘international humanitarian community’—composed, inter alia, of UN agencies, international NGOs, the International Red Cross and Red Crescent Movement, and donor states^3^—have frequently continued to justify ‘immediate’, ‘emergency’ modes of operation rather than longer‐term planning, such as through the persistent invocation of the exceptional, unexpected, sudden, and unavoidable nature of crises.

However, alternative frameworks and modes of response, including those framed around prevention, longer‐term planning, and capacity‐building, have been at the core of responses by many states and organisations, such as those characterised by the principles of SSC. Shortly after Hurricanes Irma and Jose ravaged the Caribbean in September 2017, for instance, the Cuban state deployed some 750 national health workers across the region. Their deployment was covered in the international press (Khan, 2017), but it is less widely acknowledged that Cuban doctors worked alongside Central American and Caribbean doctors who were educated in Cuba, assisting people affected by the events and their aftermath. The combined mobilisation of Cuban and non‐Cuban doctors educated in Cuba is precisely the result of the Government of Cuba having a longstanding history (since the 1960s) of engaging in forward‐planning ‘cooperation’ with Central American and Caribbean states (and others); such cooperation expanded significantly in the late‐1990s after Hurricane George hit Haiti and Hurricane Mitch claimed the lives of more than 30,000 people across Central America and displaced more than 106,000 people just in Guatemala (Fiddian‐Qasmiyeh, 2015, p. 19). In the wake of Hurricanes George and Mitch, the Cuban state not only sent internationalist medical brigades (that is, Cuban doctors) to the region, but also established a transnational education programme to train Central American and Caribbean citizens to become health workers in their own right (Fiddian‐Qasmiyeh, 2015, p. 19). The first students enrolled in the Latin American School of Medicine in Havana in May 1998, with the official aim of rendering Cuban (and other international) doctors redundant and developing sustainable models of national‐ and local‐level response for the future (Fiddian‐Qasmiyeh, 2015, p. 19).

This example reflects the extent to which capacity‐building and forward‐looking interventions have been developed and implemented historically in different parts of the world. At the same time, however, Cuba's historical and contemporary role also highlights the significance of interrogating and reconceptualising the notion of ‘the international humanitarian community’ itself. While terms such as ‘the international humanitarian community’ and ‘the international system’ continue to be widely used, as if describing fixed and internally coherent frames of reference (Telford and Cosgrave, 2007), it has nonetheless become particularly important, if not yet mainstream, to interrogate, critique, and resist who is identified, included, or excluded from these categories. Academics, policymakers, and practitioners are thus increasingly acknowledging that a plurality of ‘international communities of response’ exist, and indeed have long existed; in turn, researchers are probing why, and with what effect, ‘the Others’ of humanitarian and disaster response have been erased from the normative history of humanitarianism (Pacitto and Fiddian‐Qasmiyeh, 2013; Davey and Scriven, 2015; Fiddian‐Qasmiyeh, 2015; Fiddian‐Qasmiyeh and Pacitto, 2016).

In parallel with research programmes such as the Global History of Modern Humanitarian Action project led by the Humanitarian Policy Group at the Overseas Development Institute between 2011 and 2015 (Davey and Scriven, 2015), this author's past and ongoing work on South–South humanitarianism has explored how, why, and with what effect historical and contemporary responses have been developed and implemented by state and non‐state actors, ranging from the Cuban state since the 1950s and Libya since the 1960s, to refugee‐led responses developed in Jordan, Lebanon, and Turkey to support refugees from Syria in those countries (Fiddian‐Qasmiyeh, 2010, 2012, 2015, 2018; Pacitto and Fiddian‐Qasmiyeh, 2013). While this paper returns to the significance of Southern‐led responses to disasters below, this brief reflection simply highlights now increasing acknowledgement, in research, policy, and practice, of the importance of history *and* geography in analyses of and responses to diverse forms of disasters.

### Geographies and scales of response: roles and relationships

In addition to acknowledging the extent to which diverse actors from across the Global North and Global South^4^ have developed different forms of response to disasters over time, it is equally essential to consider the matter of the plurality of ‘communities of response’ from a multiscalar and multi‐stakeholder perspective. In this regard, research is increasingly recognising the significance both of examining the roles played by different actors, including individual, household, community, and sub‐national and national actors, as well as regional and international organisations, and of exploring the nature of *relationships* that exist within, between, and across these different responders (Pantuliano, Davey, and Kinahan, 2013).

The ‘localisation of aid’ agenda is one of the key paradigms that has been promoted as being particularly ‘innovative’ and ‘essential’ in maximising the efficiency of responses to disasters, with the international community officially asserting its commitment to supporting ‘local’ responses during and since the UN World Humanitarian Summit in May 2016; indeed, the *World Disasters Report* of 2015 documents the increased tendency for international actors to support nationally‐led strategies for disasters worldwide (IFRC, 2015). In essence, the localisation of aid agenda has been grounded in official acknowledgement of the roles played by national and regional actors in responding to disasters, and in the concomitant need for ‘the international system’ to support such ‘local’ responses in different ways. In essence, the importance here of multiscalar *analysis* in recognising the plurality of actors involved in response has been matched by a stated commitment to change modes of operation and the funding of response mechanisms—even if this shift is largely itself a reaction to the various financial and political crises that have led to pressures on the aid budgets of European and North American states. This includes the increasing trend to promote national and regional aspects of disaster management, perhaps especially, although not exclusively, in contexts of transboundary and regional disasters (Hollis, 2017).

One particular ‘regional response’ that has been repeatedly identified as providing an invaluable ‘paradigm shift’ is the UN's 3RP. Indeed, since its launch in 2014, UN documentation has repeatedly and consistently used the term ‘paradigm shift’ to describe the 3RP (3RP, 2014); notably, the extent to which the plan embodies a ‘paradigm shift’ is highlighted as one of the ‘key messages’ and ‘top‐line messages’ that officials are meant to share widely when discussing it (3RP, 2017). It is presented as being innovative—indeed, ‘a UN first’:

*The Regional Refugee and Resilience Plan (3RP) is a UN first. It represents a paradigm shift in the response to the [Syrian] crisis by combining humanitarian and development capacities, innovation and resources* (3RP, 2015, p. 6).

The 3RP has been presented, therefore, as demonstrating the ‘international community's’ commitment both to ‘forward‐looking’ policies and programmes and to supporting national and regional actors in the Global South, embodying a ‘paradigm shift’ to a ‘nationally‐led, regionally coherent strategy’ (3RP, 2014), which ‘aims to combine humanitarian assistance with development and resilience of host countries’ (ILO, 2015). However, repeatedly framing and messaging this as a paradigm shift and a UN first does not, of course, render this plan ‘a paradigm shift’, as should be evident from the earlier discussion of the long history of the humanitarian– development continuum.

This is not to say that the approach is not a welcome one (if it were to be implemented with appropriate funding), and the *3RP Progress Report* of 2015,^5^ alongside others, does helpfully centralise the importance of mainstreaming support for local municipalities and institutions into various programming activities to maximise positive outcomes and experiences among refugee *and* host communities alike in the Middle East. Indeed, the existing evidence confirms that regional‐, national‐, and municipal‐level actions and coordination *are* key to disaster response (Fiddian‐Qasmiyeh, 2016d). Evidence also confirms, though, that appropriate levels of funding and localised modes of partnership do not result from official assertions and commitments.

A further critique of the localisation framework is that although national and regional responses are often equated with ‘localised responses’, there is also a need to move towards a localisation agenda that is even more ‘local’ in nature: focusing on individuals, communities, and neighbourhoods, alongside other national and sub‐national actors, not just as ‘experiencing’ and being affected by disasters, but also as responding to these in different ways (Fiddian‐Qasmiyeh, 2018). Certainly, a fundamental challenge remains the need to explore the interconnections and diverse *relationships* between different actors at all scales (micro, meso, and macro) as processes that change across time and space. In turn, this must be completed by developing intersectionalist modes of analysis on individual and communal levels in relation to the sub‐national, national, regional, and international.

### Mass experiences and intersectionality

As suggested above, an important dimension pertaining to scales and levels of analysis is the need to reconcile ‘immediately’ responding to ‘mass’ emergency experiences and needs, with attention to intersectionality: this is because not all people, individuals, households, and communities are equally or similarly affected in a mass disaster, or have ‘standard emergency’ needs. This acknowledgement must be streamlined from the onset, even in, or before, an ‘emergency’ phase.

A clear example of the urgency of doing so emerges from the challenges of providing assistance following the Indian Ocean earthquake and tsunami on 26 December 2004, where the very definition of basic needs was demonstrated to be intimately connected to the intersecting identities of different members of the affected communities. As the tsunami hit the coastline of Banda Aceh, Indonesia, early in the morning, most people had been asleep or inside their homes, where Muslim women were not wearing the *hijab*. In this context, the United Nations Population Fund (UNFPA) rapidly acknowledged that, even if aid packages were to be delivered to the community, women (in this case, veiled Muslim women) would be unable to access them *in dignity*—here, the *hijab* was a basic needs item that was a prerequisite for Muslim women to be able access aid packages in dignity (Dakkak, cited in Fiddian‐Qasmiyeh and Ager, 2013).

In other contexts, which highlight the significance of specific belief systems, the framing of basic needs and dignity also transcends a basic need to *live* a life in dignity, pointing to the importance that different communities and individuals may accord to celebrating key rituals relating to life, and to death; in this regard, dying in dignity, and being able to bury a loved one in and with dignity can be just as important, if not more so, than what the international community often assumes to be the ‘immediate’, emergency requirements for food and shelter (see Allen and Turton, 1996; Fiddian‐Qasmiyeh and Ager, 2013). However, international agencies frequently have been reluctant to permit, or even have actively ‘resisted’ allowing, disaster‐affected people from using tarpaulin ‘officially’ designated for ‘living spaces’ for the creation of mosques or temple spaces, or to bury loved ones instead (Fiddian‐Qasmiyeh and Ager, 2013). This demonstrates a disjuncture between the assumptions held by international agencies and disaster‐affected people, and the extent to which a reconceptualisation of ‘basic needs’ requires sensitive consideration of *who the people are* who have been impacted by a given event.

As demonstrated by the tsunami in 2004, for instance, the identification of basic needs surpasses the assumption that all ‘women’ will have the same ‘basic needs’, precisely because there is no homogenised disaster‐affected ‘woman’. It is notable, of course, that it was only in the 1990s that the humanitarian system even acknowledged that millions of people impacted by disasters would have menstrual hygiene needs: sanitary materials were only supplied to women and girls as standard emergency procedure in the mid‐1990s (Sommer, 2012). Against this backdrop, it is perhaps unsurprising that it has only been relatively recently that *intersectionalist* analyses have been applied in disaster settings.^6^

As employed in the context of disaster studies, the application of an intersectionalist lens has facilitated the development of a more nuanced understanding of gendered experiences and consequences of disasters, including in a way that transcends the longstanding equation that ‘gender=women’. On the one hand, key contributions to disaster studies in the 1980s and 1990s sought to redress the earlier invisibility of women and girls in androcentric studies and policies by purposefully tracing their diverse experiences of disasters (Rivers, 1982; Enarson, 1998). On the other hand, an intersectionalist analysis starts from the understanding that experiences are constituted according to diverse intersecting, overlapping, and mutually constitutive identity markers, including gender, ethnicity, religion, class, sexual orientation, gender identity, and age, and by corresponding power structures such as patriarchy, xenophobia, Islamophobia, classism, homophobia, transphobia, and ageism. Such analyses have underlined the extent to which the relative significance of these identity markers, whether self‐ascribed or imposed by others, and related power structures shift across time and space, including in contexts of forced migration (Fiddian‐Qasmiyeh, 2014a) and other disasters (Gaillard et al., 2017).

It is through a forward‐looking, multiscalar, multi‐stakeholder, intersectionalist mode of analysis that one must move beyond technical policies framed around ‘empowerment’ and ‘promoting agency’. Far from denying the significance of people's agency and the realities of gender inequity and inequalities around the world, this provocation is offered as a way of putting a spotlight on the structural *barriers* that prevent individuals, households, communities, and states from finding and acting on their own solutions in dignity. This is to say, for instance, that it is inadequate to propose reactive and responsive measures to ‘empower’ people in contexts characterised by diverse (local, national, and international) barriers that *prevent* people from making decisions and acting upon them. Indeed, the framework of empowerment has been critiqued extensively for becoming part of a technical and technological solution that does not challenge the status quo (see Zakaria, 2017). In contrast, an intersectionalist analysis highlights precisely the extent to which certain individuals, social groups, and organisations benefit from particular disasters, while other people are exploited, marginalised, and excluded by individuals, social groups, and organisations, as well as by diverse systems of inequality and exploitation.^7^ It is by acknowledging that there are barriers and systems that prevent people from being able to act, that one can strive to find ways to remove these obstacles and enable people to find ways to live, and die, in dignity.

This is, of course, a key theme throughout disaster studies and responses: vulnerability to disasters is neither ‘natural’ nor inherent. Rather, vulnerabilities and risks are heightened by structural factors and inequalities. Intersectionality helps us to move forward with this kind of analysis, because different barriers and opportunities prevent or enable people differently. In addition to being important on an individual and communal level (Fiddian‐Qasmiyeh, 2014a; Gaillard et al., 2017), this also includes addressing the structural factors and processes that prevent lives from being lost, when knowledge and mechanisms clearly exist to do so. A recurrent theme at the fortieth anniversary conference of *Disasters* was how to implement *what we already know*, rather than continuing to find new avenues for research and practice that ultimately often end up ‘recycling’ longstanding proposals (Gaillard, 2018). Indeed, one key challenge, in the context of current and future disasters, is how to put into practice what we have collectively come to know, when resources either are not provided or are implicitly or explicitly blocked, and when responsibilities are shunned or dodged rather than upheld.

In effect, a poignant reminder with regard to the hurricanes that ravaged the Caribbean in autumn 2017 emerges in the article by Joyette, Nurse, and Pulwarty (2014) on catastrophe modelling and loss calculations in small Caribbean states vulnerable to natural catastrophes. The authors point out this ongoing vulnerability and call for more efficient ‘local governance’ to be put in place to mitigate for and to prevent disasters. However, as numerous commentators underlined following the devastation caused by Hurricanes Irma and Jose in 2017: how can efficient local governance be demanded, when local governance systems are themselves dependent on national governments that fail to oversee their ‘overseas territories’, including in this case the British Overseas Territories in the Caribbean.

In spite of ongoing challenges, ‘as a community’ of scholars and practitioners, and as *communities* and *networks* of analysis and response (who/which may or may not identify with ‘the international humanitarian community’), we are getting better at looking ‘back’ (undertaking historically grounded analysis), looking ‘elsewhere’ (towards Southern‐led responses *and* beyond the ‘international’ definition of ‘humanitarian’), and looking through different ‘lenses’ (including intersectionalist ones) to keep on looking forward.

### Interdisciplinarity and policy relevance

Indeed, studies such as those showcased in *Disasters* are multidimensional, multiscalar, and interdisciplinary, and are engaged in the ‘dual imperative’ of work that posits that ‘research should be both academically sound and policy relevant’ (Jacobsen and Landau, 2003, p. 185). There is an increasing institutionalisation in academic and donor spheres of the promotion of research that not only is ‘in conversation’ with policy and practice, but also that develops sustainable ways of working together across disciplinary and institutional silos to ensure that the world continues, progressively, to get better at anticipating, preventing, managing, and responding to disasters.

These are among the underlying principles of *Disasters*, and they have now also been strategically ‘mainstreamed’ by, inter alia, the GCRF, ‘a £1.5 million fund announced by the UK government in late 2015 to support cutting‐edge research that addresses the challenges faced by developing countries’. 8 Concerns may be raised that the GCRF could be perceived as institutionalising a dangerous instrumentalist approach—not least because it re‐designates official development assistance (ODA) funding towards research in ways that may make some researchers feel uncomfortable. Nonetheless, as part of a broader research agenda in the context of the UK, it starts from acknowledgement that although a lot has been learnt in the field of development and humanitarian studies, different and more creative ways of thinking through and about issues, and acting in response, are required. With respect to the seven Research Councils under UK Research and Innovation, this is framed as necessitating the development of more interdisciplinary approaches, including by bringing together and bridging different disciplines and schools of thought and action. The approach to interdisciplinarity equally posits that it is essential to develop multidimensional forms of understanding that push us to ask different questions, and to listen meaningfully to different voices that challenge diverse stakeholders to continue improving at responding.

This suggestion—that it is by bringing together different disciplines and approaches that we can ask different questions and ‘get better’ at addressing key global challenges— is in many ways a direct challenge to the view noted above that more research is *not* needed since ‘we’ already ‘know’ the solutions and now need to put this knowledge and these solutions into practice. However, this framework could also provide an alternative entry point to identify, and trace ways to overcome, the structural barriers that prevent these solutions from being implemented.

Here, a question for the future ‘interdisciplinarity’ of disaster studies is how one can envisage a role for the arts and humanities, not just ‘instrumentally’ (to secure grants) or as ‘seasoning’ (to what ultimately remain ‘social science’ or ‘political science’ studies), but to challenge and reconfigure what it is that we take for granted. Indeed, in a special issue of *Disasters* on the roles of historical and archival analysis, Davey and Scriven (2015, p. 113) argue that an historical approach is particularly valuable because of ‘the challenges it can pose to habitual ways of thinking and in the skills of investigation and interpretation it fosters’. The authors then advocate ‘integrating history into a more reflective attitude to change and a more adventurous and holistic approach to innovation, as opposed to simply using it to “learn lessons”’.

With this increasing space, and acknowledged need, for historical analysis in disaster and humanitarian studies, a question that emerges is whether there is also space in *Disasters* and related journals for engagement with the arts and humanities sector more broadly? For this to be meaningful in nature, I would contend that this not only entails transcending the view of the arts and humanities as offering a way of ‘better’ ‘intervening’ in disasters (for instance, to provide more efficient modes of trauma relief, as argued in Huss et al. (2015)), but also, as my colleagues and I assert within the Refugee Hosts research project,^9^ as a potential way of documenting and resisting mainstream ways of thinking about, representing, and responding to disasters per se.

## Approaching the future

With these approaches, discussions, and critiques in mind, and acknowledging the existence of multiple ‘communities of response’ rather than a singular ‘international community’ of analysis, policy, or practice, the rest of this paper reflects, with sensitivity to tempo‐spatial dynamics, on three key themes that are increasingly significant in disaster studies across all scales, levels, and directionalities: migration (including in relation to climate change); forced displacement; and Southern‐led responses.

### Migration: beyond a disaster and crisis paradigm

Perhaps counterintuitively for a reflection on a number of main themes in disaster studies, the first key trend highlighted relates to the intensifying call for analysts and practitioners to challenge and resist the (mis/ab)use of the label ‘disaster’ and ‘crisis’ when describing migration‐related phenomena. The rhetoric of ‘disasters’ and ‘crises’ has been widely mobilised since 2015—although it existed well before then of course—particularly when referring to the intersecting processes of human movement and migration. State and media discourses centralising ‘crisis’ and ‘disaster’ rhetoric have been used to justify regressive policies of control, surveillance, and, among other things, draconian border controls, the withdrawal and criminalisation of maritime rescue missions, and explicit pushbacks in direct violation of international law. In turn, when international organisations such as UNHCR have drawn on this rhetoric—ostensibly to secure humanitarian donations and attract public compassion—this has risked reinforcing despondency and fear among diverse populations (Crisp, 2017).

In contrast, historically‐ and geographically‐situated analyses have ‘debunked’ numerous elements of the discourse of ‘migration crises’, challenging claims that we are currently facing ‘unprecedented’ levels of migration or displacement worldwide, let alone confronting a ‘refugee crisis’ in Europe (Crisp, 2015; Fiddian‐Qasmiyeh, 2016b; de Haas, 2016; Ferris, 2017). On a historical level, scholars have demonstrated that the *proportion* of the global population engaging in international migration has remained remarkably stable over time. The total number of people moving has risen as the global population has increased: there were an estimated 232 million international migrants globally in 2013; this figure increased to 244 million by 2015 (UN, 2015). Massey et al (2005) and de Haas (2016, 2017), among others, have long reminded us that far from living in ‘an age of unprecedented migration’, a consistent proportion of approximately three per cent of the world's population has engaged in international migration since the 1960s. Although the proportion increased between 1990 and 2015 (2.9 per cent of the world's population were international migrants in 1990, 3.2 per cent in 2013, and 3.3 per cent in the ‘refugee crisis’ year of 2015), a core question that remains of particular interest concerns why around 97 per cent of the world's population *do not* migrate internationally.

Against this backdrop of remarkable consistency of international migration flows, it is also notable that in spite of the hypervisibility of the rhetoric of a ‘European refugee crisis’, refugees only represent between seven and eight per cent of the global international migrant population. Furthermore, despite assertions of a European refugee crisis, only about 0.4 per cent of the total European Union (EU) population was composed of refugees in 2017, a figure that was in fact higher (0.5 per cent) between 1992 and 1995 (de Haas, 2017). Furthermore, 85–86 per cent of all refugees live in developing countries, typically in states neighbouring their countries of origin. In turn, 25 per cent of all refugees reside not only in the Global South, but also in the world's least developed countries (UNHCR, 2018).

In terms of the directionalities of migration more broadly, it is notable that in 2013, the number of international migrants engaging in South to North migration (that is, people born in the Global South migrating to countries in the Global North) almost equalled the number of migrants engaging in South–South migration (that is, people born in the Global South migrating to other countries in the Global South); while definitions of who and what ‘belongs’ to the South or the North are contested, the United Nations Department of Economic and Social Affairs (UN DESA) recorded that South–South migration slightly exceeded South–North migration in 2015 : 82.4 million as compared to 81.9 million.^10^

Exact figures remain contested, yet these numbers, proportions, and percentages all point towards a particular *trend* that will remain highly pertinent for scholars, policymakers, and practitioners working in the field of disaster prevention, mitigation, and response. Migration will probably become an even more significant theme in coming years, owing, for instance, to anticipatory movements to avoid the real or assumed effects of climate change.

Despite popular assumptions that climate change will inevitably lead to a ‘crisis’ of migration (Taylor, 2017), perhaps a more accurate way of analysing this situation is using the assertion ‘no change from climate change’ (Kelman, 2014). In essence, research from around the world proves that anticipatory movements and migration as part of longer‐term planning may, or may not, be central to people's interpretations of climate‐related phenomena and processes in their localities. As is now largely mainstream in academic spheres, climate change will not *cause* migration, and it remains essential that assessments of and responses to it do not ‘depoliticise’ the challenges that affect people whose environments will indeed alter or even disappear over coming years.

Refuting deterministic and causal frameworks is not to deny that a relation between climate change and migration may exist, but rather to acknowledge that the relationship between movement, mobility, and climate events will continue to be complex and non‐deterministic. That is, there may be shifts and accentuations in migratory movements, or a total reluctance to this (Paul, 2005; Kumar Saha, 2016), as people anticipate, adapt, resist, or develop diverse coping strategies (Simatele and Simatele, 2015).

In essence, ‘disasters do not always create out‐migration’ (Paul, 2005, p. 370): in some contexts, active resistance to migration following cyclones, hurricanes, and tornados is the norm. For example, Kumar Saha (2016, p. 505) emphasised that ‘all households want[ed] to avoid migration’ following Cyclone Aila in coastal Bangladesh in 2009, and yet structural conditions that prevent livelihoods from being re‐established may mean that ‘some form of widespread migration is inevitable after a disaster such as [Aila]’. Even in the light of this widespread reluctance, migration may ‘have the potential to serve as a key adaptive response to environmental events, as evidenced by the improved economic conditions of a substantial number of the migrated households’ (Kumar Saha, 2016, p. 505). As such, when disaster‐affected households do migrate, this may lead to improved socioeconomic outcomes, rather than migration itself resulting in losses or crises on different levels. Indeed, migration and mobility are normal, everyday features of livelihood strategies around the world (Carruth, 2017), as demonstrated by longstanding research with members of communities with pastoralist and nomadic backgrounds and livelihood strategies, even in contexts of forced displacement (Fiddian‐Qasmiyeh, 2014b; Carruth, 2017).

Hence, in some settings, migration *can* be an ‘adaptive strategy to climate variability’ (Simatele and Simatele, 2015), while in others (such as the example from Bangladesh cited above), people may express an active reluctance to migrate, to be relocated, or to be resettled elsewhere. This aversion is a key trend in the Global North and Global South, as evidenced in relation to Hurricane Sandy in 2012 (Bukvic and Owen, 2017) and, more recently, as echoed in many local responses in the Caribbean and United States before, during, and after Hurricanes Irma and Jose made landfall, in which people from across the whole spectrum of socioeconomic backgrounds remained reluctant to move.

Concurrently, *not* being able to move, including because of structural barriers, and physical ones such as the erection of border walls and the establishment of enforced ‘safe zones’ in Syria and elsewhere, can be indicative of an existing or emerging crisis. In essence, immobility is often a marker or indictor of particular risk on individual and communal levels alike (Fiddian‐Qasmiyeh, 2016b). While a search of the *Disasters* archive in 2017 only revealed three articles that included the word ‘immobility’, it will become a key theme of disaster studies research, both with respect to internal and international migration, echoing the interest in immobility in migration and mobility studies (Hannam, Sheller, and Urry, 2006).

### Forced displacement: urban, protracted, and overlapping displacements

As noted, involuntary immobility is a fundamental and yet invisible dynamic within processes of forced displacement (Lubkemann, 2008). The latter, in contrast, is hypervisible, especially during the early onset of mass displacement, and even more so when displaced people reach not only European television screens but also European borders (Fiddian‐Qasmiyeh, 2016b). However, despite mass migration being at the core of popular, political, academic, and policy responses to forced migration, the implications of three intersecting trends related to forced displacement—that is, its urban, protracted, and overlapping nature—will require further consideration in coming years.

Urban and protracted displacement became extensively researched processes from the 1990s. This attention and the development of international policies, such as UNHCR's Policy on Alternatives to Camps, were heralded by many observers as a significant paradigm shift, challenging international humanitarian organisations’ outdated—and many would argue inhumane, inefficient, and inadequate (Harrell‐Bond, 1986; Malkki, 1995)—camp‐based ‘care and maintenance’ policies. Such accounts regularly indicate the extent to which refugees’ residence in non‐camp, urban settings has *increased* numerically; the challenges of supporting refugees who live in towns and cities for protracted periods of time thus warrant more attention and the development of new, context‐specific modes of analysis and forward‐looking responses that merge short‐term humanitarian and longer‐term development elements.

However, some classic examples from *Disasters* help one to situate the ‘urban turn’ in displacement studies, by reminding us that in the late‐1970s ‘too much attention was being paid to refugees in urban areas’ (Pantuliano, 2011). This overemphasis on refugees in urban settings in essence led Chambers (1979) to focus on the *differential* experiences of rural and urban refugees and to acknowledge the particular problems faced by rural refugees. With UNHCR having seemingly encountered major operational challenges in addressing the needs and rights of refugees in urban environments since the 1990s—with its first urban refugee policy being critiqued from diverse angles, including by Crisp (2017) (see also Crisp, Morris, and Refstie (2012))—one can interpret the title of the UN's ‘Adapting to an Urban World’ project as being as much about the need for refugees to ‘adapt’ to urban settings as it has been about the need for UN agencies, including UNHCR, to ‘adapt’ to urban spaces. Nevertheless, as suggested above, this has in fact perhaps been a ‘return’ to the urban ‘bias’ that had prevailed not so long ago and had prompted Chambers to look *beyond* urban refugees in the late‐1970s.

Echoing the commitment of Chambers (1979) and Pantuliano (2011) to centralising the heterogeneous needs of refugees and the displaced around the world, and the need to balance a focus on different spaces of arrival and settlement (camp, rural, urban, and everything in between), it is important also to concentrate on the *relationships* and *interactions* that exist between different groups of refugees in a diversity of spaces, and indeed, to complement a focus on ‘refugee–refugee relationality’ (Fiddian‐Qasmiyeh, 2016a) with a focus on relationships between heterogeneous members of different groups of refugees and different groups of hosts across time and space. These relationships are particularly significant in light of the third major trend set out above, which is intimately related to the urban and protracted nature of displacement: overlapping displacement.

A great deal of academic and policy attention has been accorded to urban and protracted displacement, but very little research has been undertaken on the nature and implications of overlapping displacement, including with regard to the relationship between refugees and local communities. The term ‘overlapping’ is used here to refer to two tempo‐spatial dynamics. First, refugees and internally displaced person (IDPs) have often personally and collectively experienced secondary and tertiary displacement. This is the case for thousands of Sahrawi and Palestinian refugees who left their refugee camp homes in Algeria and Lebanon, respectively, to study or work in Libya before being displaced by the outbreak of conflict there in 2011, as well as for Palestinian and Iraqi refugees who had originally sought safety in Syria only to be displaced once more by the conflict in that country (Fiddian‐Qasmiyeh, 2012, 2015). Second, refugees are increasingly experiencing overlapping displacement in the sense that frequently they physically share spaces with other displaced people. For example, it is reported that Turkey hosts refugees from more than 35 countries of origin, Lebanon from 17, Kenya from 16, Jordan from 14, Chad from 12, and Ethiopia and Pakistan from 11 (Crawford et al., 2015).

The implication of these intersecting processes it that, precisely because displacement is increasingly urban and protracted, refugees share spaces for longer periods of time both with local host communities, and with other displaced people themselves. This means, inter alia, that, over time, refugee groups often become members of the communities that subsequently offer protection and support to other groups of displaced people.

The significance of overlapping displacement is perhaps particularly evident when considered in relation to the drive for longer‐term programming and going beyond linear approaches to disaster response, as noted by Twigg (2015) in his introduction to the virtual special issue of *Disasters* on recovery: ‘The old, simplistic, notions of disasters as a *temporary interruption* in development, and recovery as a *return to pre‐disaster normality*, are clearly no longer viable’ (emphasis added). The process of overlapping displacement indicates precisely not only the extent to which people continue to experience ongoing forms of vulnerability and precariousness over time—or indeed, increased vulnerability as displacement becomes increasingly protracted (Barbelet and Wake, 2017, p. 24)—but also the degree to which the ‘new norm’ (if not the ‘new normal’) for many people may be to be displaced or affected by a crisis again and again, either individually or as families and members of communities that have experienced displacement on more than one occasion in their lifetime, or as people who remain displaced and then become ‘hosts’ of newly displaced people.

A contemporary example is that of Palestinian refugees in the urban Baddawi refugee camp in north Lebanon, who have resided there since the 1950s and who have ‘hosted’ refugees arriving from Syria since 2011. These refugees include not only displaced Syrians, but also Palestinian and Iraqi refugees who had been living in Syria at the outbreak of the conflict and who have found themselves refugees once more (Fiddian‐Qasmiyeh, 2012, 2015). In the context of Baddawi, Palestinians are simultaneously refugees and hosts, and urban camps are spaces that are shared between not only different generations of refugees, but also refugees with different nationalities and countries of origin. Furthermore, this is also not the first time that the Baddawi camp and its refugee inhabitants have welcomed ‘new’ refugees, as it also hosted more than 15,000 ‘new’ Palestinian refugees who were internally displaced from the nearby Nahr al‐Bared refugee camp when it was destroyed during fighting in 2007. With an estimated 10,000 refugees from Nahr al‐Bared still residing in the Baddawi camp, these ‘internally displaced refugees hosted by refugees’ have become part of the established Baddawi community hosting ‘newly’ displaced refugees from Syria (Fiddian‐Qasmiyeh, 2016e).

Such processes of overlapping displacement and shared spaces indicate the importance of examining refugee–refugee relationality, requiring that research, policies, and practices transcend and critique the implications of the assumption that citizen‐host communities are ‘affected’ by refugees, or that ‘citizens’ ‘support’ or ‘reject’ displaced people. Instead, it is essential to examine carefully the relationships that exist, emerge, and change over time and space between different groups of people who have been directly and indirectly affected by and involved in complex emergencies and disasters, including protracted displacement.

Highlighting the relational nature of displacement, and destabilising the assumption that refugees are hosted by citizens, is evidently not to idealise the encounters that characterise refugee–refugee encounters, since these are also often framed by power imbalances and processes of exclusion and overt hostility between members of new and established refugee communities. However, rather than viewing these tensions as inevitable, certain policies and programmes may activate resentment and insecurity among hosts, and there is an increased need to fulfil the aforementioned commitment to implement development‐oriented programmes that aim to support refugees and host communities (as is ostensibly at the core of the 3RP and dozens of initiatives since at least the 1960s). In the context of overlapping displacement and refugees hosting refugees, these tensions may be the result of the uneven development and implementation of programmes for different ‘generations’ of refugees and for refugees according to their country of origin. This is particularly visible in Baddawi, whose long‐term residents have received limited (and increasingly insecure) assistance from the United Nations Relief and Works Agency for Palestine Refugees in the Near East (UNRWA) since the 1950s, while new arrivals from Syria have the potential to receive support from an expanding range of inter/national organisations, including UNHCR (Fiddian‐Qasmiyeh, 2016e, 2019).

A challenge that remains for researchers, policymakers, and practitioners in acknowledging the widespread reality and implications of overlapping displacement is to engage simultaneously and meaningfully with the agency of refugees and their diverse hosts as active responders in disasters, while also recognising the challenges that characterise such encounters. At a minimum, new programmes and policies must avoid re‐marginalising established refugee communities that are hosting newly displaced people; at best, with appropriate attention (and political will) they can be sensitive to supporting the needs and the rights of all people affected by displacement, whether they are hosting or being hosted.

### Southern‐led responses: beyond instrumentalisation

Far from passively waiting for externally supplied assistance, communities, families, households, individuals, states, and regional organisations across the Global South have been responding to disasters every year, decade, and century. In this regard, the case of refugees hosting refugees can be examined using the framework of ‘locally provided aid’, as well as one of a number of ‘Southern‐led’ responses.

Recognition of the roles of diverse Southern actors in disaster situations has been enhanced, and indeed ‘institutionalised’, via the aforementioned ‘localisation of aid’ agenda. On the one hand, we must remain concerned about the instrumentalisation of Southern actors and the extent to which the localisation agenda may be a way for Northern states to shift resources and responsibilities to Southern actors, or simply to withdraw from international responsibilities without sharing promised funding and resources (Fiddian‐Qasmiyeh, 2015, 2018, 2019). On the other hand, it affords an opportunity to acknowledge the extent to which Northern approaches are limited while Southern‐led initiatives can have major advantages. For instance, Wamsler and Lawson (2012, p. 28) argue that ‘Northern cities could learn some valuable lessons from the rich range of comparatively more advanced local coping strategies used to face disaster risk in the Global South’. In turn, the United Nations Development Programme (UNDP)'s ‘headline story’ in its *Human Development Report 2013*, entitled *The Rise of the South: Human Progress in a Diverse World*, is that: ‘The South needs the North, and increasingly the North needs the South’ (UNDP, 2013, p. 2). Furthermore, beyond incorporating local/Southern actors into the ‘international system’, or identifying the transferability of ‘lessons learned’ from the South to the North, there is also a prime opportunity to reconsider the role of ‘local’ actors in and from the Global South in developing *alternative* modes of response that at times can work alongside or explicitly challenge ‘normative’ Northern‐led responses (Fiddian‐Qasmiyeh, 2016b, 2018).

There is of course a major paradox inherent in the localisation of aid agenda outlined above, in so far as it aims to ‘support’ local responses precisely by institutionalising them within the broader paradigm and parameters established by the ‘international system’. In this context, the localisation of aid agenda can be seen as promoting a particular form of North–South relations, in which Northern states have recognised Southern actors and increasingly are mobilising them to ‘share the burden’ (precisely by ‘keeping the burden’ in the South) in undertaking assistance and protection activities (Fiddian‐Qasmiyeh, 2015). The mainstreaming of support for Southern‐led initiatives by UN agencies and Northern states is especially paradoxical with respect to ‘SSC’, as the latter was purposefully developed in the era of decolonisation as a necessary means to overcome the exploitative nature of North–South relations, and historically has been associated with the Non‐Aligned Movement and anti‐colonial and anti‐imperialist struggles (Fiddian‐Qasmiyeh, 2015, 2018; Fiddian‐Qasmiyeh and Daley, 2018).

As such, a focus on Southern‐led responses must transcend identifying and offering (certain forms of) support to specific *actors* from the Global South; instead, it invites us to consider what role diverse modes of SSC may play in terms of responding to disasters, and what role the *principles* and *modalities* of formal and informal SSC might have in reconceptualising existing and formulating new or hybrid forms of response, including those that challenge structural inequalities. It is this *relationality* between diverse actors across the Global South, at all scales, levels, and directionalities, and the divergent principles, motivations, and modes of action, which remain to be explored in detail (Fiddian‐Qasmiyeh, 2018).

Of course, while the *Human Development Report 2013* noticeably fails to address SSC with respect to conflict‐induced displacement, Southern states *have* worked individually and collectively to develop regional initiatives to protect people affected by disasters, including displacement,^11^ well before the ‘paradigm shift’ announced with reference to the 3RP (Fiddian‐Qasmiyeh, 2015, 2018). There is, as noted, a long history of Southern‐led responses in contexts of disaster relief, including responses officially driven by the principles of SSC. These include Cuba's aforementioned involvement in Central America and the Caribbean, explicitly to enhance the region's ‘self‐sufficiency’ and to reduce dependence on externally provided assistance/protection.

However, while SSC has been officially perceived as being central to development and responses to environmental hazards, until recently it has frequently been seen, in principle, to be incompatible with responding to conflicts and conflict‐induced displacement. Here, SSC in disaster response can be understood as a means of providing assistance to a disaster‐affected *state* to strengthen its ability to offer assistance to its own citizens on its own territory after an ‘environmental disaster’. In contrast, delivering assistance in conflict and displacement situations in which the state is either involved as a belligerent party or has demonstrated little or no political will to offer protection to its population, could be understood as a breach of the South–South principles of respect for national sovereignty and non‐interference, when such involvement does not take place at the explicit behest of the state itself. Yet, historical and contemporary analyses demonstrate a diversity of Southern‐led modes of assistance to and protection of refugees and IDPs, including those funded, designed, and implemented by states and regional organisations.

In spite of this ‘evidence,’ it remains the case that the actual and potential role of SSC (whether in its principles, aims, or modes of operation) in conflict and displacement situations has remained almost ‘unimaginable’, including by the very UN agency that holds the South–South ‘portfolio’: UNDP.^12^ For instance, UN agencies and international NGOs often assume that SSC can only take place when ‘time’ is available, with humanitarian situations excluded almost a priori. This is clearly reflected in the following quotation from a senior UNDP employee interviewed by Omata (2018, p. 276):

*Making South–South initiatives requires a long‐term vision and strategic planning. Before making a deal, it involves numerous negotiations between involved actors and UNDP…. I know UNHCR staff need to respond quickly to emergencies to save people's life. These emergencies usually emerge in an unpredictable way. Such situations are not conducive to the modalities of South–South partnerships*.

As such, while UNDP has an established track record of promoting SSC in the context of development, it has often been assumed by and with regard to UN agencies that SSC is incompatible with ‘humanitarian’ work because it necessitates long‐term planning, while UNHCR needs to operate from one hour to the next. Of course, we know this is not the case overall, and that diverse Southern actors—regional organisations, states, sub‐national actors, communities, and individuals—will continue to play key roles in responding to different disasters, including conflict‐induced displacement.

With reference to human displacement, for instance, SSC must be more meaningfully explored in existing and new and emerging displacement situations precisely because conflict and displacement‐related ‘crises’ frequently are predicted or even ‘announced’ weeks, months, and years in advance, and precisely because displacement is increasingly protracted in nature. UNHCR is indeed making (very slow) headway in institutionalising modes of SSC, such as through its promotion of the ‘solidarity resettlement scheme’ between the Middle East and specific solidary Latin American countries (Fiddian‐Qasmiyeh, 2015, 2018; Omata 2018; c.f. Cantor, 2018). However, there is also a need to complement such approaches, by recognising the extent to which forward‐looking initiatives developed under the remit of SSC have not only existed historically around the world, but have legacies that still echo to date. For example, from the 1970s to the present, Cuba's international scholarship system has offered secondary‐ and tertiary‐level education to refugee youth, including Namibians, Palestinians, Sahrawi, and South Sudanese (Fiddian‐Qasmiyeh, 2010, 2015); among other things, this has meant that many Palestinian and Syrian students who trained in Cuba to become doctors and surgeons between the 1970s and 2000s have been providing medical assistance to people displaced within and from Syria, including Iraqis, Kurds, Palestinians, and Syrians (Fiddian‐Qasmiyeh, 2018).

Given the reality of such responses, one could argue that it is in conflict situations that Southern‐led responses (whether through modes of SSC or via the localisation agenda) have met particular resistance. This is because they directly question ‘international’ humanitarian principles and institutionalised modes of response, and because they have the potential to challenge the current system in ways that would require fundamental changes to its very foundation.^13^

## Concluding remarks: looking forward

There are different ways of imagining and implementing responses to disasters, including models based on principles of SSC and horizontal learning that can spawn longer‐term responses to emerging and protracted displacement scenarios. By highlighting a series of examples that are framed officially or informally as modes of SSC, it has not been the intention of this paper to idealise such responses (for critiques, see Fiddian‐Qasmiyeh, 2015, 2018). Rather, it has aimed to exemplify, first, the extent to which diverse actors are, and have long been, involved in responding to disasters, and, second, the extent to which their underlying principles and objectives may differ from, and challenge, those of the more mainstream ‘members of the international humanitarian system’. While Global North actors may continue to reject many of these interventions for being ideological and political rather than categorising them as modes of ‘humanitarian’ assistance—Cuba, for instance, often has been depicted as engaging in ideologically motivated forms of ‘disaster diplomacy’^14^—a key issue that remains to be explored further is how people affected by disasters themselves experience, enact, respond to, and *conceptualise* these different processes and modes of response. This is essential since people affected by disasters do not merely ‘experience’ or ‘respond’ to them, but also are everyday theorists who conceptualise, negotiate, and resist different forms of action and inaction.^15^

Sahrawi and Palestinian refugees who studied in Cuba in the 1990s and 2000s before returning to work in their home‐camps in Algeria and Lebanon, respectively, for instance, referred repeatedly to the scholarship programme via reference to a combination of ‘ideology’, ‘politics’, ‘humanitarianism’, and ‘human values’ (Fiddian‐Qasmiyeh, 2015). Ultimately, they maintained that Cuba's programme for refugees *is* ‘humanitarian’ in nature, but they offered different perspectives regarding the *balance* between these different dimensions, noting, implicitly and at times explicitly, the ways in which they overlap or are in tension. Hence, rather than describing the programme as a humanitarian programme per se, the interviewees offered remarkably similar humanitarian ‘qualifiers’, describing Cuba's scholarship programme as having ‘a humanitarian *component*’, ‘a humanitarian *dimension*’, a ‘humanitarian *aspect*’, and ‘humanitarian *ingredients*’; other interviewees stated that it is ‘a *mainly* humanitarian system’, with ‘humanitarian *elements*’, and ‘shares its humanitarian *message* in spite of the [US] embargo [on Cuba]’ (cited in Fiddian‐Qasmiyeh (2015)).^16^

Acknowledging peoples’ ‘experiences’ of disasters, and indeed the ways in which local stakeholders ‘respond’ to disasters, has been key both to improving operational responses in the field, and to recognising the agency of people who are vulnerable to disasters owing to diverse structural and social inequalities. In turn, acknowledging people's needs and their ‘agency’ in a tempo‐spatially sensitive, intersectionalist manner must now be a foundational premise for analysis and action alike; this is essential precisely to establish the ways in which diverse identity makers and structures of oppression and opportunity interact to enable or prevent different modes of action and being. In this regard, it is particularly urgent to continue interrogating the relationship between what external analysts assume are, and should be, ‘the’ priorities of people affected by disasters, and what different people affected by conflict, disasters, and displacement may themselves prioritise, and which systems and structures may be limiting their ability to live meaningful and dignified lives. The examples of the veil as a basic need for dignity in the aftermath of the Indian Ocean tsunami in 2004, and of disaster‐affected people using ‘shelter tarpaulin’ to create mosque or temple spaces or to bury loved ones, all demonstrate the need to consider, in an intersectionalist manner, whose priorities and basis needs are prioritised by which actors and which structures oppress or marginalise them.

However, once that foundational approach is in place, it also becomes essential to go beyond looking at people's ‘experiences’ and even focusing on the ways that people ‘act’ in response to displacement. Instead, or rather catalysed by that foundational approach, it becomes vital to centralise more systematically both how people ‘conceptualise’ their own situations, positions, and responses, and to concentrate intently on identifying and challenging the diverse structural barriers, including cultural, economic, political, and social ones, which prevent specific people in specific disasters from living, and dying, in dignity.

In the context of highlighting key trends for disaster studies, the final section of this paper proposed the value of analysing historical and contemporary forms of SSC on diverse scales (rather than an instrumentalisation of Southern *actors* via the ‘localisation of aid’ agenda), and of critically engaging with the *principles* and *modalities* of SSC. Among other things, the SSC framework may be a useful lens through which to examine disaster response, as it has, from its emergence, blurred and/or combined rather than inscribed institutional and programmatic distinctions between long‐ and short‐term responses: the very term ‘cooperation’ has the potential to encompass both development *and* humanitarian initiatives, thereby potentially enabling us to transcend the impasse of the recycled ‘development–humanitarian continuum’ (Fiddian‐Qasmiyeh, 2015, 2018). In turn, South–South actions and principles have distinctive spatialities, directionalities, and imperatives of response at their core, including particular attention to developing modes of challenging and redressing structural inequalities that ultimately ‘create’, or at least magnify, vulnerabilities to diverse disasters.

Highlighting these potentialities is not a matter of idealising them, but rather a means of proposing that further research is required. This is needed first to understand better how different actors, on different scales and levels, experience, perceive, *and* conceptualise how, why, and with what effect different forms of response are implemented by Southern responders (including in formal and informal SSC). Second, it is required to trace, resist, and challenge the diverse structural barriers that prevent the development of meaningful responses that meet individual and collective needs and rights around the world.

## Endnotes

1 This part of the paper is informed by the author's ongoing project entitled Local Community Experiences of and Responses to Displacement from Syria (also known as Refugee Hosts (http://www.refugeehosts.org)), funded by the Arts and Humanities Research Council (*AHRC*) and the Economic and Social Research Council (*ESRC*) (AH/P005438/1) and supported by the Leverhulme Trust (PLP‐2015–250).

2 This part of the paper draws on the author's ongoing project entitled Southern Responses to Displacement from Syria: Views from Lebanon, Jordan and Turkey (http://www.southernresponses.org), which has received funding from the European Research Council (ERC) under the European Union's Horizon 2020 research and innovation programme (grant agreement no. 715582). While beyond the scope of this paper, this project and the author's research more broadly involves a critical analysis of conceptualisations and taxonomies of ‘the South’, and proposes the value of Southern, anti‐colonial, decolonial, and postcolonial theories to enrich understanding of, and ways of responding to, the world (see Fiddian‐Qasmiyeh and Daley, 2018).

3 Other conceptualisations of the members of ‘the international humanitarian community’ or ‘the international disaster community’ exist (as discussed below), yet these are the key entities that typically are associated with this ‘international community’ (Telford and Cosgrave, 2007).

4 For a critical discussion of the different ways of conceptualising ‘the South’ and ‘South–South encounters’, see Fiddian‐Qasmiyeh and Daley (2018).

5 For more information, see http://www.3rpsyriacrisis.org/wp-content/uploads/2015/06/3RP-Progress-Report.pdf (last accessed on 8 January 2019).

6 Intersectionality as a concept and analytical framework originated in the 1980s and early‐1990s. Crenshaw (1991) first developed it as a means of exploring and explaining the overlapping experiences of oppression and marginalisation faced by African‐American women by virtue of their gender and race in a society characterised by everyday, institutionalised patriarchy and racism.^.^

7 This is, of course, also argued in political economy approaches to disaster studies, including Keen (1994) and de Waal (2018).

8 For more information on the GCRF, see https://www.ukri.org/research/global-challenges-research-fund/ (last accessed on 9 January 2018).

9 See http://www.refugeehosts.org (last accessed on 9 January 2018).

10 See Chikanda and Crush (2018) for more on South–South migration.

11 For instance, Southern states and regional organisations have developed dynamic national and regional legal frameworks to protect refugees and IDPs, including the Organisation of African Unity's 1967 *Convention Governing the Specific Aspects of Refugee Problems in Africa* (Omata, 2018) and the 1984 *Cartagena Declaration on Refugees* (Cantor, 2018). In turn, the world's first ‘international’ convention on internal displacement was drafted by the African Union and entered into force in 2009: the *African Union Convention for the Protection and Assistance of Internally Displaced Persons in Africa* (for more information on Southern‐led responses to displacement, see Fiddian‐Qasmiyeh (2018)).

12 UNDP was home to the ‘Special Unit for Technical Cooperation among Developing Countries’ between 1974 and 2004; this was renamed the ‘Special Unit for South–South Cooperation of the United Nations Development Programme’ in 2014.

13 I would like to thank an anonymous peer reviewer for encouraging me to develop further this line of analysis here.

14 For a critical discussion of ‘disaster diplomacy’, see Kelman (2007).

15 See Fiddian‐Qasmiyeh (2016c) on the relationship between, on the one hand, viewing refugees and stateless people as ‘experiencing’ displacement and a lack of legal protection, and, on the other, recognising that refugees and stateless people *conceptualise* their own situations and those of others.

16 For an evaluation of this scholarship programme and the application of tempo‐spatial, multiscalar, and intersectionalist analyses, see Fiddian‐Qasmiyeh (2015).
